# An Overview of the Current State and Perspectives of Pharmacy Robot and Medication Dispensing Technology

**DOI:** 10.7759/cureus.28642

**Published:** 2022-08-31

**Authors:** Asmaa R Alahmari, Khawlah K Alrabghi, Ibrahim M Dighriri

**Affiliations:** 1 Computer Science Department, Faculty of Computing and Information Technology, King Abdulaziz University, Jeddah, SAU; 2 Department of Pharmacy, Al Qurayyat General Hospital, Al Qurayyat, SAU; 3 Department of Pharmacy, King Abdulaziz Specialist Hospital, Taif, SAU

**Keywords:** pharmacy robot, pharmacy automation, pharmaceutical, robotics, medication dispensing

## Abstract

It has been widely reported that a large number of patients die from cases of errors in the issuing of medication prescriptions. These cases occur due to a wide range of things, but the common denominator in all of the cases is humans. A hospital pharmacy has a very critical task, especially with growing patient numbers. The increasing number of prescriptions needed to be filled daily reduces the amount of time that the staff can use to focus on each individual prescription, which may increase the human error ratio. The need for robotic-assisted pharmacies is arising from here to distribute drugs to eradicate or substantially reduce human error. The pharmacy robot is one of the most significant technologies that play a prominent role in the advancement of hospital pharmacy systems. The purpose of this review paper is to cover the pharmacy robot concept and the published literature reporting on pharmacy robot technology as one of the most important applications of artificial intelligence (AI) in pharmacology. Although the outcomes of the impact of the pharmacy robot have been increasingly beneficial in overall improvement, staff morale, and functionality of pharmacies, there are still mechanical errors occurring. The errors, in turn, require human intervention. The key takeaway from this study is that robots or machines cannot replace human duties in their entirety. This in turn means that those human interventions will have an impact on the workflow and throughput.

## Introduction and background

Introduction

The aim of a hospital pharmacy is to provide patients with the prescribed medication when the medication is scheduled according to the professional’s instructions. However, this is not an easy task, especially with growing patient numbers. Although patient safety and care are the ultimate and possibly only priorities, the impact of human error in the process of issuing medication to patients can be, at times, deadly. These errors occur in various ways, such as the incorrect dosage being issued or the medication not being issued at the correct time. Nonetheless, all of these can and do have serious consequences. In addition, this is a major logistical concern as the pharmacy delivers drugs by various dispensing methods and delivery routes to all the hospital units [1,2].

There are many different types of technologies available inside a hospital pharmacy framework that work to enhance patient safety by reducing prescription mistakes and missing drugs. The pharmacy robot is one of the most significant technologies that play a prominent role in the advancement of hospital pharmacy systems [1-5]. The pharmacy robot is almost flawless in medication administration, and the correct algorithm allows the application of the five rights as published in a study [6]. These rights refer to the only way to avoid errors by ensuring the correct patient received the correct medication and dosage in the correct administration at the correct time.

Using robots ensures a considerable decrease in the time, costs, and production of overall waste in pharmaceutics and other biological research fields [2,7]. The time needed to prepare the prescription is also one of the significant advantages of using robotic technologies [7]. Besides, robotics reduces the percentage of medication errors [8].

Even though robots can provide unattended operations for the handling of pharmaceutics, they need the attention of an operator as machine errors still occur, albeit rarely. Thus, a large concern is that artificial intelligence (AI) will displace humans in their duties, and although there are ethical questions when implementing AI in the healthcare sector, it is inevitable that human intervention will continue. These human interventions do, however, affect both the workflow and throughput. Human intervention could take the form of an operator who needs to direct, load, or unload products. Mechanical issues, defined as errors within the system, continue to occur, which then require intervention from an operator. Mechanical errors were recorded as varying problems, such as material problems, which included vials or fluid bags not falling within the weight parameters, misshapen needles, or the inability for the robot to successively grip or hold a vial due to manufacturing defaults in the assembly of the robot. Other errors occurred due to manufacturing changes whereby measurements of syringes or other items were incorrect. Barcode and vial recognition failures were also reported, and this is due to the limitations of the robot's being able to overcome multiple simultaneous versions of a task. All of these errors required the intervention of the pharmacist or technician in order to verify necessary data, adjust parameters, or repair manufactured defaults [9]. The purpose of this article is to review pharmacy robot technology as one of the most important applications of AI in pharmacy.

Background

What Are Artificial Intelligence and Pharmacology?

In simple terms, AI is the ability of a system, mostly computers, to act intelligently and therefore perform tasks that would normally require human intervention. There are many things that power these systems; they can be simple things like rules, machine learning, or even deep learning [10]. Pharmacology refers to the chemicals used in the treatment of illness and disease [11].

What Is a Robot?

According to various sources of literature, a robot is considered an intelligent agent, either virtually or mechanically. This agent can then carry out tasks either under the supervision of guidance, typically via remote control, or automatically. An automaton, which is the common name for an autonomous robot, refers to a robot that can undertake various tasks in environments that are either structured or not. The robot is able to perform these tasks without human supervision. Robotics has seen substantial advances over the past years leading to its increased applications in numerous real-world complications including automated industrial manufacturing, healthcare and medical robots, and self-driving vehicles [12]. Today’s society has seen the use of these robots across various applications, fields, and sectors. Examples include the transportation, medical, military, and banking sectors, to name a few. Relevant to this study is the use of robots within the pharmaceutical sector; these robots that are usually functioning autonomously can be programmed and configured in order to make the distribution of prescription drugs more effective and efficient, therefore reducing the need for human intervention and, in turn, reducing the risk of human error [13].

Robots make use of map-making algorithms in order to create a map of their environment. These algorithms allow the robot to have an accurate display of its environment, which is essential for the effectiveness of the application. These maps are used in the same way that humans use maps. The robots use the maps for guidance to “see” their environment. The accuracy of these maps is important as it forms the basis of the practical applications needed to be carried out by the robots [13].

As with all technology, the aim of this kind of technology is to ensure effective, efficient, and accurate application within the setting. This in turn means that the pharmacy robot will be able to take the art of traditional medical dispensing into the next era. The benefits of this particular application include increased productivity; a medical dispensing fault-free environment; pharmaceutical operations that are effective, safe, and secure; shortened patient "waiting period," and a germ-free and safe environment [14].

Pharmacy Robot's System Architecture

**Figure 1:**
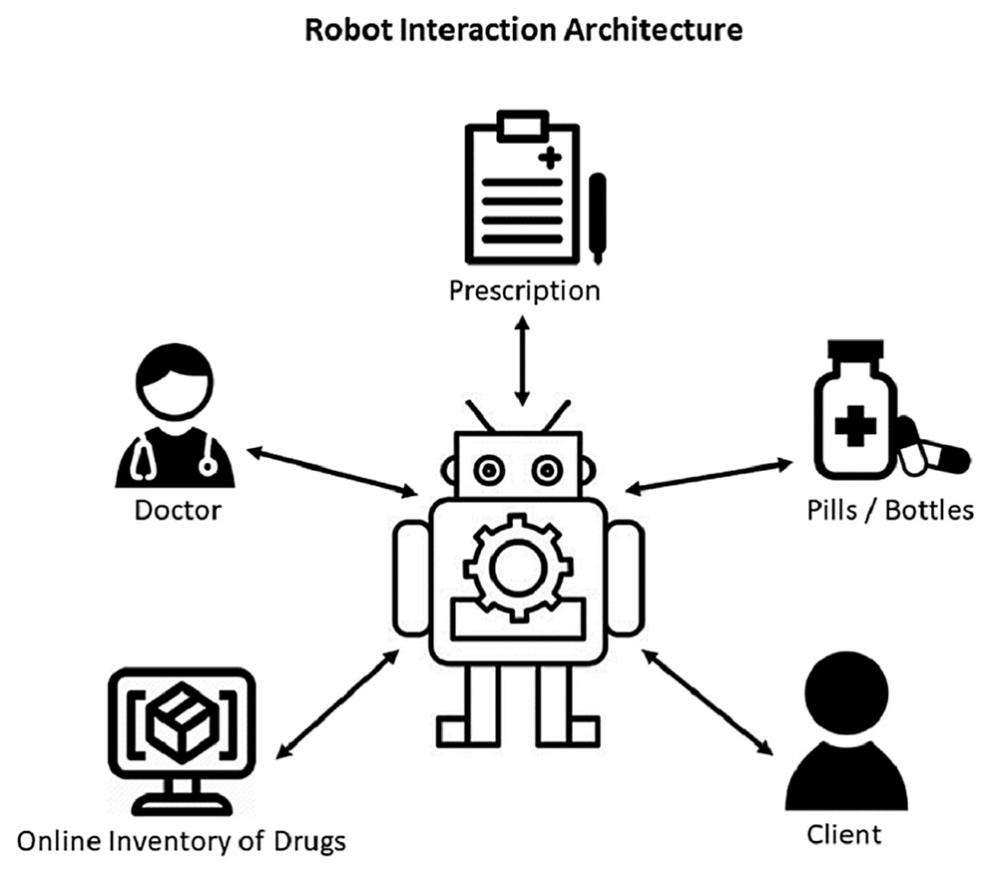
General robot interactions

For efficiency, the best fit across multiple areas is usually custom. From the view of the system architecture, each application, robot, or system is custom designed and built based on the needs and functions of the system. All aspects involved within the application are carefully considered when an automation system is built. In this section, in order to understand the system architecture, we will explain in detail the pharmacy robot system architecture by giving an example of a pharmacy robot that was introduced by some authors [13]. As we see in Figure 1, the robot communicates autonomously and directly with a physician, assistant, or intermediary. The robot then scans the inventory of stock in order to assess the availability of the prescribed medication. When the prescribed medication is available, the robot will fill the container with the medication and store the filled container. This is all done by the robot referencing and interacting with its environment using the internal map created with the map-making algorithm. The specific robot in this article makes use of simultaneous localization and mapping (SLAM). When the patient or representative arrives to collect the prescription, the robot will validate the identity of the individual receiving it. The patient or representative then presents the prescription, and the robot will then issue the prepared medication over to the patient. As mentioned above, this specific robot makes use of AI and SLAM in order to interact with a variety of things. Not only does SLAM allow the robot to have a clear and accurate map of its surroundings, but it also allows the robot to update the map when needed, keeping an accurate and updated “vision” of the current locations of inventory [13].

**Figure 2:**
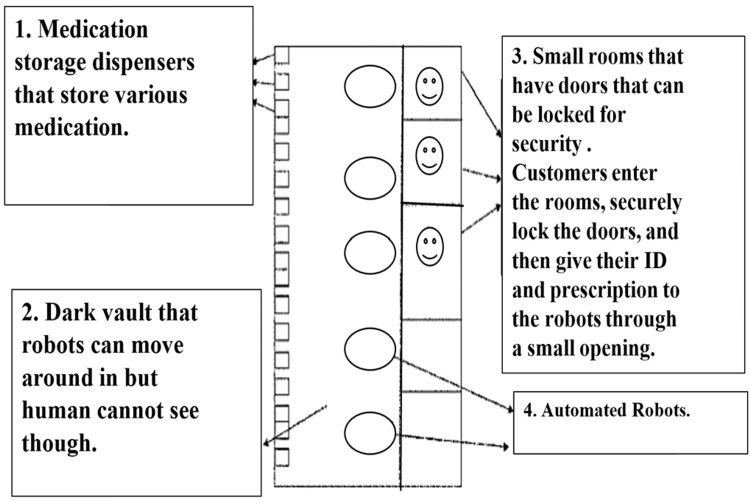
Architecture of the pharmacy area

Figure 2 illustrates the physical architecture of the pharmacy area; this is often referred to as an embodiment. Multiple customers may enter and choose an available room. These rooms are individually secure as they can be locked from within [13]. Over and above this, the vault, which is the store for a variety of medications and dispensers, can be controlled according to specific requirements, such as light and temperature. This allows for the control and reduction of the deterioration of various medicines that require refrigeration, etc. This setup allows the servicing of multiple people at one time [13].

**Figure 3:**
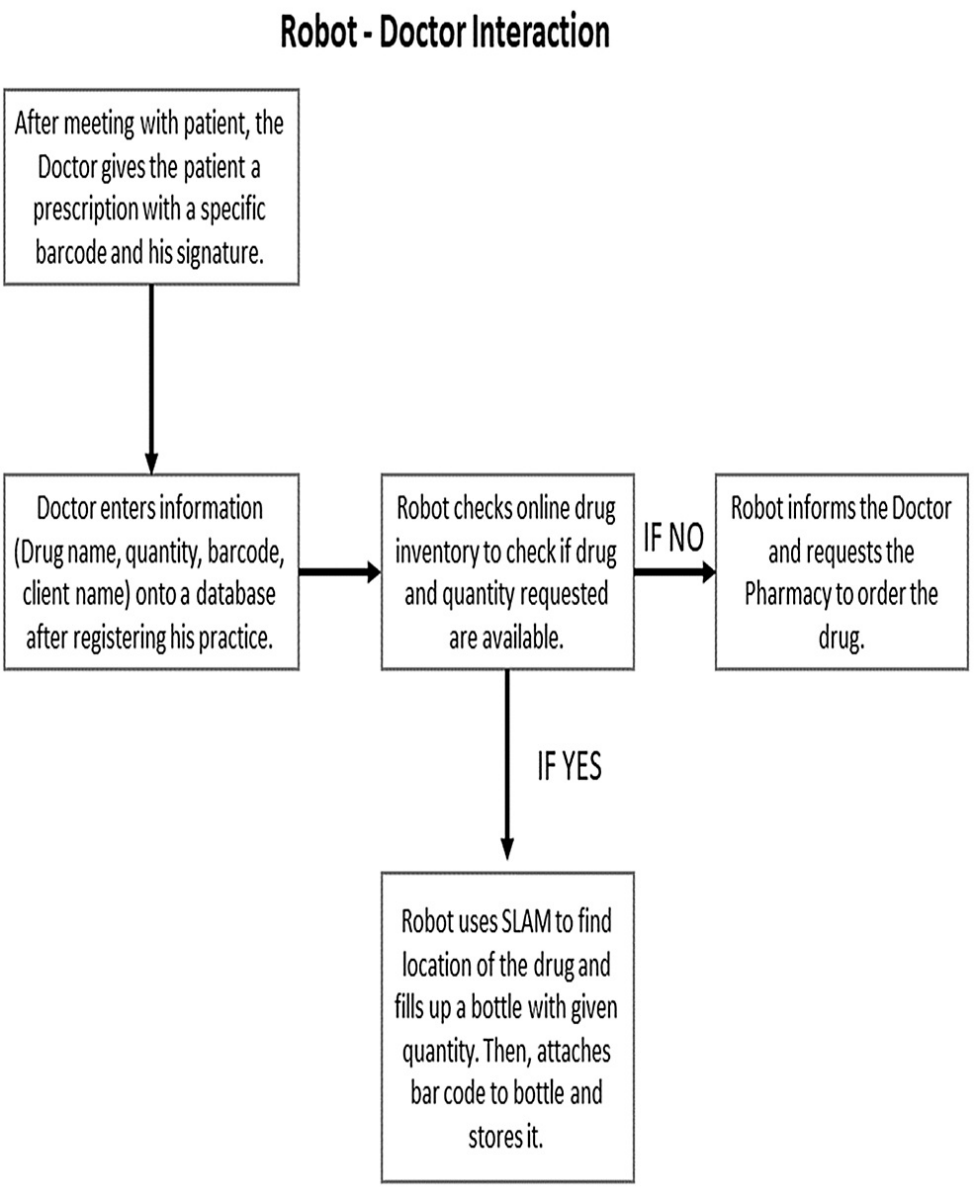
Robot's interactions with medical professionals

Figure 3 is to clarify the interactions between the robot and medical staff such as doctors or assistants like nurses [13]. The patient is consulted and examined by a doctor. The patient is then issued a prescription from the doctor. This prescription will contain an authentication mark (barcode, signature, etc.) [13].

**Figure 4:**
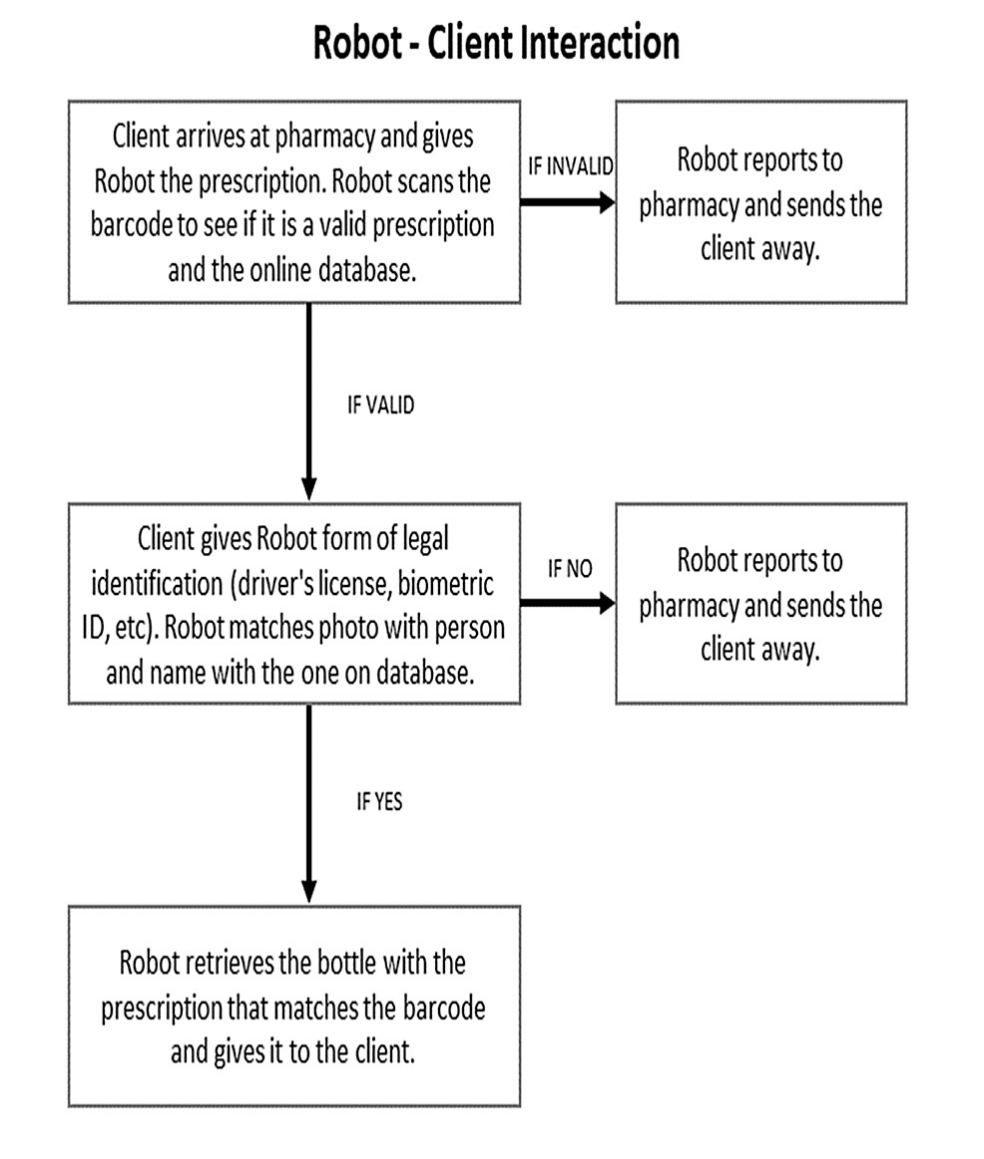
Robot's interactions with clients

Figure 4 clarifies the interactions between the robot and clients or patients [13]. If the prescribed medication is in stock, the robot authenticates the prescription by accessing the patient’s file on the network [13].

**Figure 5:**
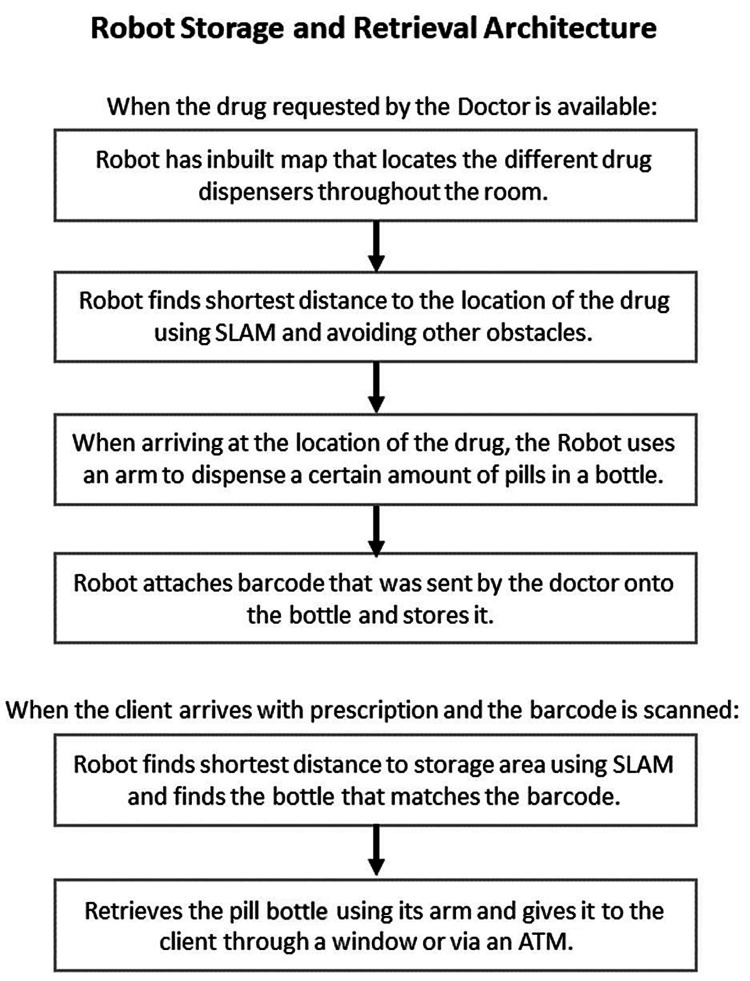
Architecture of retrieval and storage by the robot

The retrieval and storage of functions of the robot could be as follows in the sequence as shown in Figure 5 [13].

**Figure 6:**
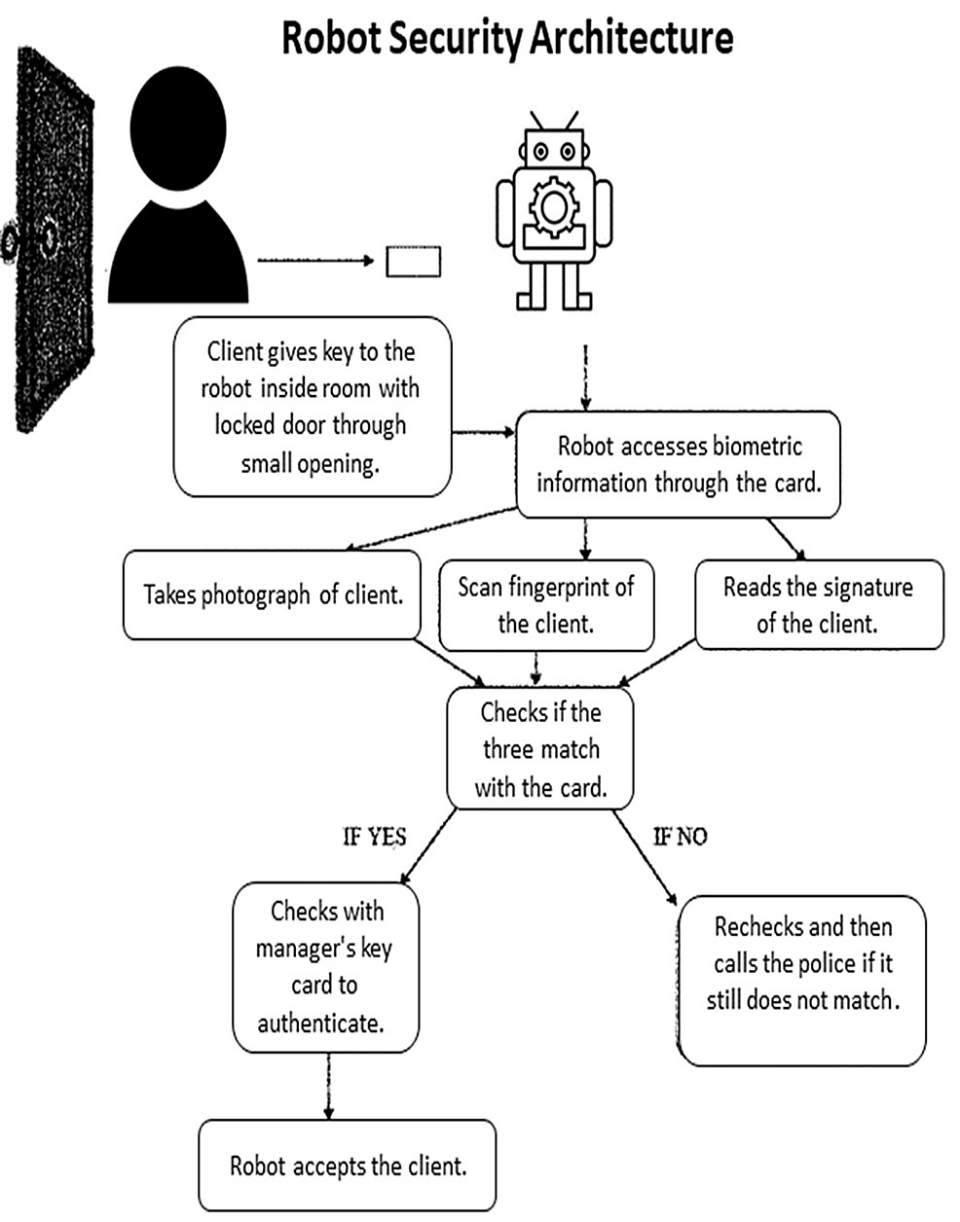
Robot security architecture and biometric identification

Patients are verified and validated using their biometrics on file. Once complete, the patients can access their medication as shown in Figure 6 [13].

## Review

The objective of the current review paper is to cover the published literature reporting on pharmacy robot technology as one of the most important applications of AI in pharmacology. The exploratory search process covered the most relevant organizations in the pharmacology community. The search was conducted using the IEEE Computer Society Digital Library, Association for Computing Machinery (ACM) Digital Library, ScienceDirect, SpringerLink, and Google Scholar. These search engines cover the majority of published pharmacology studies as of April 20, 2020. These search results were screened and filtered to include studies of any design, which reported an outcome measure of interest that was related to the technology of the robot pharmacy. Publications in English that were published in 2010 to date were included. The title, abstract, and full publications were used to identify citations of interest. Once the full review of the publications was completed, several eligible publications were selected for inclusion in our review.

Why a pharmacy robot?

According to www.youhaverights.com, in the United States, it is estimated that 30 million pharmacy errors occur annually. An estimated 7,000 patients die from these cases of errors and thousands of severe complications each year. These errors occur due to the misinterpretation of the prescription by the pharmacist with regard to medication name or dosage, to name a few. The reasons for the occurrence of these errors usually seem to stem from the overworked pharmacy employees. These employees operate under extreme pressure and have ever-increasing workloads. The increasing number of prescriptions needed to be filled daily reduces the amount of time that the staff can use to focus on each individual prescription. The most common pharmacy errors are incorrect dispensing of medications, incorrect dosage being administered due to the failure of staff to instruct consumers on specific medication usage, and incorrect delivery and preparation of IV (intravenous) drugs to patients in hospitals [15].

So, the need for robotic-assisted pharmacies arises from here to distribute drugs to eradicate or substantially reduce human error. The advantages of robotics in pharmacy are to improve and add to the current patient care and therefore increase revenue, improvement in the overall functionality and operation of pharmacies, and effective management and storage of medication as well as patient data. Overall enhanced productivity leads to increased dispensing speed and, therefore, decreased patient waiting time. It allows for the decentralizing of pharmacy services and the evolution of ward-based medicine management. This allows the elimination or significant decrease in the number of errors and contamination. Therefore, using barcoding and photo verification increases patient safety and decreases the risk of litigation. This improves dispensing efficiency and enables the re-engineering of pharmaceutical services, which leads to maximum utilization of space in hospital pharmacy departments [16-18]. The use of AI is an improvement of the traditional pharmaceutical dispensing system. Although we have seen the evident improvement and efficiency that technology adds to various systems, such as the reduction or absence of human error, as with anything that is not perfect and has been created by man, it would be unwise to not take into account the disadvantages that technology presents. These disadvantages include the following: computer literacy and staff competencies may impact their ability to manage or program the dispensing robot. This could lead to extensive input of training over a significant period of time. The dispensing robot requires accurate programming as incorrect programming may lead to errors. As with most technology, there are sizable startup costs, maintenance costs as well as the need for continuous software updates. It is well known that with the evolution of technology, systems can be considered out of date quite quickly, which will then require updating. Although robots will bring increased and improved efficiency and productivity, the redundancy of pharmacists will never occur. Where there is a limited pharmacist and patient interaction, it might make patients reluctant to use the service. These dispensing robots rely on computer programs that can fail at any time. The maintenance and upgrade of software as well as the connection of the dispensing robot to the pharmacy’s system could be timely and will require meticulous detail in order to avoid any repercussions and errors.

Examples of pharmacy robot

The following are some of the most widely known unit dose drug automation systems in the world. These are Pyxis and Rowa Speedcase automation systems. Their advantages and disadvantages can be summarized as follows in Table 1 [1,19,20].

**Table 1:** Some examples of pharmacy robots

References	Name of Robot	Year	Advantages	Disadvantages
[19,20]	Pyxis automation system	2019	Workload moved from the pharmacy to the clinic floors. Fingerprint verification ensures that only authorized individuals have access to the medicine. Allows keeping track of stock. Receives all drug-related reports. Ensures that the proper medicine is available at the right moment.	Unable to track lots as a unit dose. Mistakes could occur as a result of reinstalling the returned medication. The inability of checking the expiration dates of the same medications in the same drawer. Issues with data transmission. Barcode verification of tablet medications is performed only at the bedside. The inability of preventing medication mistakes completely.
[1]	Rowa Speedcase system	2008	Decreased department costs, medication mistakes, and patient drug distribution times. Improved service efficiency. Stock control is relatively easy.	This technology is unable to package unit doses and deliver medications to the clinic.

A comparison between robot pharmacies and traditional pharmacies

Pharmacy robots are mechanical devices that conduct planned, sophisticated, and repetitive manipulations that mimic human behavior without continual input from a human. As part of their treatment, the majority of patients require medication. Various technology-based solutions, such as computerized physician order entry (CPOE) and patient barcoding (BC) systems, have been implemented in hospitals to increase patient safety. According to current evidence, documenting, dispensing, and giving drugs to patients are all high-risk steps in taking medicine [21].

Automated methods have been introduced in many pharmaceutical distribution systems to address the risks of manual dispensing and administration systems. In addition, there is a lot of pressure to cut expenditures and reallocate time from manual distribution. Putting these resources toward greater clinical work has been a success. New methods and procedures must be developed to reduce patient wait times, provide drug therapy information to physicians and pharmacists, and increase productivity by automating administrative tasks [22].

van Doormaal et al. reported that manual medication evaluation revealed 57 pharmaceutical overdose errors and 143 therapeutic errors, 46 of which were drug-drug interactions. A total of 297 safety alerts regarding overdose and 365 safety alerts involving drug-drug interactions were generated using CPOE and basic clinical decision support systems. The clinical rules yielded 313 safety alerts, accounting for 39% of all overdoses and treatment mistakes discovered in the manual assessment. The patients in 23% of the warnings generated by a clinical rule required a medication adjustment, as indicated by the manual evaluation [23].

Amodeo et al. reported that the median error detected during reconstitution, dilution, and final therapy of medications made by the I.V. Station® was less than 5%, with narrower error ranges than drugs prepared manually. Unlike the manual technique, the I.V. Station® consumed fewer materials, lowered expenses, cut preparation time, and streamlined the medicine process as the number of preparations increased [24]. Table 2 shows a comparison between robot pharmacies and traditional pharmacies.

**Table 2:** A comparison between robot pharmacies and traditional pharmacies

	Advantages	Disadvantages
Robot pharmacies	Robot pharmacies can help with medicine organization, expiration date monitoring, and prescription preparation. Stock control and replacement orders are automated, which saves time for technicians. Finally, the likelihood of drug errors is reduced. The central hospital pharmacy and the ward can employ these automated systems for decentralized dispensing. These systems simplify drug dispensation by the nurse on the ward and improve drug storage until the patient requires it. As a result, there is a decreased likelihood of medication errors, better drug usage, and less time spent by nurses and pharmacists, which may be redeployed to patient care [25]. To assure accuracy, robotic dispensing devices can count pills and bottles, label prescription vials, store hundreds of drugs, and access patient data. Moreover, they act similarly to vending machines in that they may dispense medications to patients based on their prescriptions. This may encourage more frequent monitoring, which can help with drug tapering and medication misuse prevention [26]. Pharmacy automation technologies assist pharmacists in avoiding mistakes and contamination. Robotic systems keep each different drug in separate cells to prevent cross-contamination. Many automation systems have a picture verification capability that digitally records each pill it fills, which increases the patient's safety while minimizing the risk of liability for pharmacies. Moreover, the patient's demands are met faster, boosting their satisfaction [27]. Robotic dispensing machines protect patients by reducing the likelihood of human error. Moreover, these systems are now capable of handling huge daily prescriptions. Most robotic systems incorporate several protections to ensure that the correct medication is administered to the right patient at the precise dosage every time [28].	The disadvantages of robotic pharmacy include high initial costs, mainly due to required facility improvements. Because robotic pharmacy technologies are expensive, the losers of this technology are the employees who lose their jobs to balance the expense of the system [29]. Robotic pharmacies can eliminate human errors; it is crucial to remember that machines make mistakes. Aside from the cost, robotic pharmacies need solutions for items like blood derivatives, flammables, and cytotoxic medications. Considering the substantial capital investment necessary, these limitations make robotic pharmacists an undesirable alternative for many health services [30]. Automated dispensing machines are controlled by computer programs that can fail unexpectedly. Updating the software and connecting the unit to the pharmacy's own record management system can take time and should be done carefully to avoid future failures. Additionally, robotic pharmacies can be costly; they entail significant startup and maintenance costs, and some computer software requires continuous upgrades. As a result, hefty payments are required each time the product is updated [21].
Traditional pharmacies	Pharmacists talk to patients, educate them about their drugs, and detect drug-drug interactions and food-drug interactions. Additionally, they answer patients' questions to improve the quality of their lives and provide primary preventative care, such as vaccines and health screenings [31,32]. Pharmacists are progressively becoming integral members of the caregiving team, fostering greater collaboration in modern healthcare than ever before. Each day, pharmacists are required to perform clinical and preventative care functions in addition to the typical dispensing activities. Physicians increasingly depend on pharmacists to achieve a more clinical role along the caregiving continuum [33].	Errors in medications dispensed happen because of look-alike or sound-alike medications, interruptions, occupational stressors, and complex prescriptions [34]. However, heavy workloads, insufficient staffing, and employee stress have been linked to dispensing errors [35]. The pharmacist had to hand count, fill, cap, and label patients' prescriptions, which took a lengthy time. This resulted in pharmacists having significantly less time to devote to patient service and clinical obligations as members of the caregiving team. Additionally, this repetitious process performed manually posed a risk to patient safety. Regardless of how vigilant and safety-concerned pharmacy personnel are, medication-dispensing errors continue to be a problem [36,37].

Obstacle avoidance by hospital ward inspection robots in a complex environment

Laser radars, vision modules, ultrasonic radars, and infrared radars are the principal sensors used to identify obstructions. There are two types of laser radar: 2D and 3D laser radars. Because of the narrow detection range of infrared radars, they have little value for warning ahead of time. Similarly, ultrasonic radars have little use for warning ahead of time due to ultrasonic reflection and poor orientation performance [38].

Detection of Obstacles

With the laser sensor carried on its own body, the robot identifies dynamic and static impediments and establishes the laser sensor's detection model. Using a multilayer perception strategy based on the potential field exclusion approach, the robot judges the obstacle's perception layer in terms of different distances between the robot and the obstruction and then pushes itself to execute different mobility strategies. The design of the threshold value between each layer is dependent on the robot's running speed, deceleration, acceleration, the current electric amount of power, and the corridor's size [39,40].

## Conclusions

AI applications have left visible marks in many different fields. With robots, hospital pharmacies will increase their performance, increase prescription filling levels, improve counting accuracy, minimize drug errors, increase safety, ensure adherence to patient doses, automate their supply chains, and avoid delays in supplies and stock outages. Besides, robots can also help them minimize costs as they do not need to hire additional staff to support the extra load at peak times. The advantages are apparent, but what this means for humans and their sustainability is a concern. If robots are able to perform better, there will be no need for humans, and large-scale unemployment could occur. The famous late Stephen Hawking said, “This may mean the end of the human race." It would therefore be essential to create AI to work with humans rather than independently. AI can ensure accuracy within the parameters of the task, and humans will be able to have a broader reach when making decisions. Clinical decisions can be improved with AI and thus drive further research. The important goals of our future research will be to cover more studies related to pharmacy robots and medication dispensing technologies. We are also interested in comparing the studies with analysis and critical evaluation. It would be interesting to see how smart hospitals embrace this growing trend in the future.

## References

[1] Goundrey-Smith S (2013). Pharmacy automation. Information Technology in Pharmacy.

[2] Svirsko AC, Norman BA, Hostetler S (2020). Standardizing pharmaceutical delivery to reduce pharmacy costs while simultaneously reducing missing doses. IISE Trans Healthc Syst Eng.

[3] Alam S, Osama M, Iqbal F, Sawar I (2018). Reducing pharmacy patient waiting time. Int J Health Care Qual Assur.

[4] Flynn EA, Barker KN (2006). Effect of an automated dispensing system on errors in two pharmacies. J Am Pharm Assoc (2003).

[5] Angelo LB, Christensen DB, Ferreri SP (2005). Impact of community pharmacy automation on workflow, workload, and patient interaction. J Am Pharm Assoc.

[6] (2022). The five rights: a destination without a map | institute for safe medication practices. https://www.ismp.org/resources/five-rights-destination-without-map.

[7] Lin AC, Huang YC, Punches G, Chen Y (2007). Effect of a robotic prescription-filling system on pharmacy staff activities and prescription-filling time. Am J Health Syst Pharm.

[8] (2022). Watch robots transform a CA hospital. https://www.npr.org/sections/money/2015/05/27/407737439/watch-robots-transform-a-california-hospital.

[9] Yaniv AW, Knoer SJ (2013). Implementation of an i.v.-compounding robot in a hospital-based cancer center pharmacy. Am J Health Syst Pharm.

[10] Sabharwal A, Selman B (2011). S. Russell, P. Norvig, artificial intelligence: a modern approach, third edition. Elsevier.

[11] Kenakin TP (2019). A Pharmacology Primer: Techniques for More Effective and Strategic Drug Discovery, Fifth Edition. Elsevier.

[12] Haidegger T (2019). Autonomy for surgical robots: concepts and paradigms. IEEE Transactions on Medical Robotics and Bionics.

[13] (2022). Pharmacy automation using autonomous robot. https://patents.justia.com/patent/11200979.

[14] Oswald S, Caldwell R (2007). Dispensing error rate after implementation of an automated pharmacy carousel system. Am J Health Syst Pharm.

[15] Singhai M, Singhai AK, Verma K (2021). Applied mathematics for pharmaceutical problems using robotics as assistive tools for learning: a comprehensive review. Jurnal Teori dan Aplikasi Matematika.

[16] Khader N, Lashier A, Yoon SW (2016). Pharmacy robotic dispensing and planogram analysis using association rule mining with prescription data. Expert Syst Appl.

[17] Dalton K, Byrne S (2017). Role of the pharmacist in reducing healthcare costs: current insights. Integr Pharm Res Pract.

[18] Cresswell K, Cunningham-Burley S, Sheikh A (2018). Health care robotics: qualitative exploration of key challenges and future directions. J Med Internet Res.

[19] (2022). Pyxis MedStation™ ES System. https://www.bd.com/en-uk/products/medication-management/point-of-care/pyxis-medstation-es-system.

[20] Kiran B, Sencan N (2019). A general evaluation on drug distribution and automation systems in pharmacy. International Conference on Data Science.

[21] Boyd AM, Chaffee BW (2019). Critical evaluation of pharmacy automation and robotic systems: a call to action. Hosp Pharm.

[22] Hynniman CE, Lamy PP (1967). Outpatient pharmacy automation. Am J Heal Pharm.

[23] van Doormaal JE, Rommers MK, Kosterink JG, Teepe-Twiss IM, Haaijer-Ruskamp FM, Mol PG (2010). Comparison of methods for identifying patients at risk of medication-related harm. Qual Saf Health Care.

[24] Amodeo I, Pesenti N, Raffaeli G (2019). Robotic therapy: cost, accuracy, and times. New challenges in the neonatal intensive care unit. Front Pharmacol.

[25] Kuiper SA, McCreadie SR, Mitchell JF, Stevenson JG (2007). Medication errors in inpatient pharmacy operations and technologies for improvement. Am J Health Syst Pharm.

[26] Lagrange FJ, Lagrange JV (2021). Process performance of a new liquid medication dispensing robot [IN PRESS]. Eur J Hosp Pharm.

[27] Capilli M, Enrico F, Federici M, Comandone T (2022). Increasing pharmacy productivity and reducing medication turnaround times in an Italian comprehensive cancer center by implementing robotic chemotherapy drugs compounding. J Oncol Pharm Pract.

[28] Seger AC, Churchill WW, Keohane CA (2012). Impact of robotic antineoplastic preparation on safety, workflow, and costs. J Oncol Pract.

[29] Sng Y, Ong CK, Lai YF (2019). Approaches to outpatient pharmacy automation: a systematic review. Eur J Hosp Pharm.

[30] Momattin H, Arafa S, Momattin S, Rahal R, Waterson J (2021). Robotic pharmacy implementation and outcomes in Saudi Arabia: a 21-month usability study. JMIR Hum Factors.

[31] Sanii Y, Torkamandi H, Gholami K, Hadavand N, Javadi M (2016). Role of pharmacist counseling in pharmacotherapy quality improvement. J Res Pharm Pract.

[32] Youmans SL, Schillinger D (2003). Functional health literacy and medication use: the pharmacist's role. Ann Pharmacother.

[33] Freeman CR, Abdullah N, Ford PJ, Taing MW (2017). A national survey exploring oral healthcare service provision across Australian community pharmacies. BMJ Open.

[34] James KL, Barlow D, Burfield R, Hiom S, Roberts D, Whittlesea C (2010). A study of unprevented dispensing incidents in Welsh NHS hospitals. Int J Pharm Pract.

[35] James KL, Barlow D, Burfield R, Hiom S, Roberts D, Whittlesea C (2011). Unprevented or prevented dispensing incidents: which outcome to use in dispensing error research?. Int J Pharm Pract.

[36] Bateman MT Jr, McCarthy C, Alli K (2020). Linked pharmacist-provider new patient visits in primary care. Am J Manag Care.

[37] Roumeliotis N, Sniderman J, Adams-Webber T (2019). Effect of electronic prescribing strategies on medication error and harm in hospital: a systematic review and meta-analysis. J Gen Intern Med.

[38] Mon Y-J (2015). Machine vision based sliding fuzzy-PDC control for obstacle avoidance and object recognition of service robot platform. J Intell Fuzzy Syst.

[39] Cu W, Zhang P (2020). Dynamic obstacle avoidance of vision sensor mobile robot based on prior knowledge. IEEE.

[40] Fuzhong W, Haibo L, Fashan Y (2008). Obstacle avoiding strategy of mobile robot via binocular stereovision. IEEE.

